# Dual function of MDM2 and MDMX toward the tumor suppressors p53 and RB

**DOI:** 10.18632/genesandcancer.120

**Published:** 2016-09

**Authors:** Jesús Hernández-Monge, Adriana Berenice Rousset-Roman, Ixaura Medina-Medina, Vanesa Olivares-Illana

**Affiliations:** ^1^ Instituto de Física, Universidad Autónoma de San Luis Potosí, Av Manuel Nava No 6 Zona Universitaria CP 78290. SLP, México

**Keywords:** MDMX, MDM2, RB, p53

## Abstract

The orchestrated crosstalk between the retinoblastoma (RB) and p53 pathways contributes to preserving proper homeostasis within the cell. The deregulation of one or both pathways is a common factor in the development of most types of human cancer. The proto-oncoproteins MDMX and MDM2 are the main regulators of the well- known tumor suppressor p53 protein. Under normal conditions, MDM2 and MDMX inhibit p53, either via repression of its transcriptional activity by protein-protein interaction, or via polyubiquitination as a result of MDM2-E3 ubiquitin ligase activity, for which MDM2 needs to dimerize with MDMX. Under genotoxic stress conditions, both become positive regulators of p53. The ATM-dependent phosphorylation of MDM2 and MDMX allow them to bind *p53* mRNA, these interactions promote p53 translation. MDM2 and MDMX are also being revealed as effective regulators of the RB protein. MDM2 is able to degrade RB by two different mechanisms, that is, by ubiquitin dependent and independent pathways. MDMX enhances the ability of MDM2 to bind and degrade RB protein. However, MDMX also seems to stabilize RB through interaction and competition with MDM2. Here, we will contextualize the findings that suggest that the MDM2 and MDMX proteins have a dual function on both p53 and RB.

## INTRODUCTION

The p53 and retinoblastoma (RB) proteins are two key tumor suppressors. Mutations in one or both are found in all human cancer tumors and both have been extensively studied as potential therapeutic targets in drug development programs. p53 is a transcription factor in which converge many cellular stress pathways such as oncogene activation, hypoxia, DNA damage, and endoplasmic reticulum stress, to induce different biological cell responses such as cell cycle arrest in G1 or G2, DNA repair, senescence, or even apoptosis [1]. As a result of these features, p53 is named “the guardian of the genome”. In a normal healthy cell, p53 remains at a very low concentration, but after cellular stress, its level increases. Due to the importance of p53, it has to be very tightly controlled; MDM2 and MDMX are known as the main regulators of this tumor suppressor protein and are altered in many human cancers [2–7]. Their negative influences toward p53 have been extensively characterized. Nevertheless, both are also positive regulators of p53 expression; the cellular conditions are the key determinant in whether p53 is up- or down- regulated [8–11].

Conversely, RB is implicated in many cellular processes such as cell cycle regulation, differentiation, chromatin remodeling, and mitochondria-mediated apoptosis [12–14]. Loss of RB function can occur through mutation in the *RB* gene itself, by hypermethylation of the *RB* promoter, by binding of viral proteins such as E7 of the human papillomavirus or E1A of the adenovirus, or through post-translational modifications with tumor- associated kinase activity [15–17]. Given the importance of the functions of RB in the cell, its activity and levels are also tightly regulated. Phosphorylation is the most well-characterized post-translational modification of RB, particularly phosphorylation of Cdk/cyclin complexes, which plays a role in RB inhibition during cell cycle control [18]. However, only a few E3 ubiquitin ligase proteins have been reported as regulators of RB: during virus infection, cullin 2 is able to target RB for degradation via the human papillomavirus protein E7; SCF^SKP2^ is also able to target RB via the Epstein-Barr EBNA3C protein; and under non-viral infection conditions, RB is ubiquitinated by NRBE3 and MDM2 [19–23]. The regulatory activity of MDM2 and MDMX proteins on the tumor suppressor RB is becoming the subject of focus. We yet know that MDM2 is able to degrade RB through two different mechanisms, while MDMX, on the one hand, helps MDM2 to degrade RB, and on the other, it is also able to avoid the MDM2-dependent degradation of RB. However, the conditions under which each event occurs have not yet been clarified.

To illustrate the relevance of the interplay between MDM2/MDMX and RB and p53 in human cancers, we will give an overview of progress in the field, the similarities and differences in the regulation of these important tumor suppressors through MDMX and MDM2, and finally analyze the crosstalk between the p16^Ink4a^/RB/ E2F and the p14^ARF^/p53/MDM2/MDMX pathways.

### MDM2 in the regulation of RB and p53

**Figure 1:**
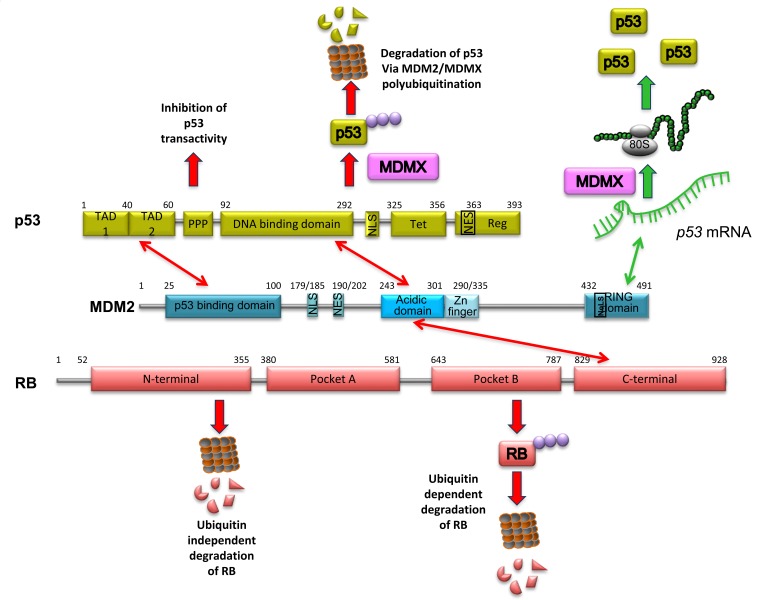
Schematic representation of MDM2, p53 and RB The different domains of each proteins are indicated, the sites of interaction between the proteins and the physiological consequences of each interaction.

p53 and MDM2 interact directly through the N-terminal regions of both proteins (Figure 1). It has been proposed that this first interaction in the N-terminal promotes a second contact that involves the acidic domain of MDM2 and the DNA-binding domain of p53 [24–26]. The interaction between p53 and MDM2 induces proteasomal degradation of p53 via polyubiquitination [27, 28], hence the reputation of MDM2 as the main negative regulator of p53. The effect described above is responsible for keeping p53 at very low levels, under normal cellular conditions. Less well-known is the fact that MDM2 can also act as a positive regulator of p53. Under genotoxic stress conditions, ATM phosphorylates MDM2 at Ser395, near to its RING domain, allowing the protein to expose a site for the *p53* mRNA interaction that enhances p53 translation [9, 11]. Using either doxorubicin or etoposide to induce DNA damage in the H1299 cell line, it is possible to see an MDM2-dependent enhancement of transfected p53 levels (Table 1), a phenomenon that helps to explain the fact that MDM2 is one of the first p53-transcribed genes. Thus, MDM2 has a dual role toward p53, and the key to allow it to switch from negative to positive p53 regulator is the cellular environmental condition.

**Table 1:** Effects of MDM2 and MDMX on the tumor suppressors p53 and RB

MDM2	Positive effect	Negative effect	Binding site
Effect on p53	[8, 9, 11] Under DNA damage: after ATM phosphorylation, MDM2 enhances p53 translation.	[27, 89] Under normal conditions: p53 degradation via ubiquitination and inhibition of transactivity of p53.	[25, 26, 90] p53 hydrophobic pocket residues 18 to 23 (N-terminal region); MDM2 p53 binding domain (N-terminal region), a secondary interaction site between DNA binding domain of p53 and the acidic domain of MDM2.
Effect on RB	NR*	[32] Under MDM2 overexpression: RB is degraded via ubiquitination. [35] Under non-stress conditions: RB is degraded independent of ubiquitination. [43] Under DNA damage: degradation after p38-dependent phosphorylation of RB.	[30] MDM2 residues 254 to 264 (ac. domain); RB residues 785 to 803 (the C-terminal pocket) [32].
MDMX			
Effect on p53	[10] Under DNA damage: after ATM phosphorylation, MDMX is an RNA chaperone to enhance p53 translation.	[50, 91]Under normal conditions: inhibition of transactivity of p53 and ubiquitination and degradation via MDM2.	[47, 92] p53 hydrophobic pocket (N-terminal region); MDMX p53 binding domain (N-terminal region), a secondary interaction site between DNA binding domain of p53 and the acidic and RING domains of MDMX.
Effect on RB	[54] Under normal conditions: stabilization of RB by competition with MDM2.	[55] Under normal conditions: degradation in an MDM2-dependent manner.	[55] MDMX residues 432 to 481 (C-terminal RING domain); RB C-terminal pocket. [54] RB C-terminal pocket; MDMX ΔC-terminal.

* NR: not reported

RB and MDM2 also interact directly; in this case, the acidic domain (residues 254 to 264) of MDM2 is responsible for binding with the RB C-terminal region (residues 785 to 803) (Figure 1). The same region of RB is involved in the interaction with the E2F1 transcription factor [29–31]. An important implication of the interaction between RB and MDM2 is the reduction of RB levels. In 2005, two different reports showed that MDM2 promotes RB degradation. On the one hand, Uchida et al. found that MDM2 promotes RB ubiquitin-dependent degradation *in vivo* in HEK293, NIH3T3, HCT116, MEF, SRB1, and U2OS cell lines [32]. This effect was suppressed by the presence of p14^ARF^, as has previously been shown for p53 [33, 34]. They also observed that this effect is selective toward RB, since neither p107 nor p130 underwent MDM2-dependent ubiquitination even when they interacted with MDM2 [32]. In the same year, Sdek et al. determined that MDM2 promotes proteasomal degradation of RB, but in an ubiquitin-independent manner [35]. Using the U2OS, C33A, H1299, SJSA-1, SAOS, and ts20 cell lines, they carried out a series of experiments where they detected ubiquitinated forms of p53 but not of RB. They confirmed these results using the ubiquitin mutants K47R and K48R, which both block polyubiquitination, and even in the presence of these mutants they were able to observe the RB degradation. Finally, they observed the formation of a triple complex between MDM2, RB, and C8, a subunit of the 20S proteasome. RB is able to interact directly with C8, but the presence of MDM2 enhances this interaction, promoting RB proteasomal degradation independent of ubiquitination [35]. Likewise, other proteins such as p53 and p21 are degraded by ubiquitin- dependent and independent mechanisms [36–41]. The above-described facts seem to confirm that MDM2 is a key negative regulator of RB (Table 1). However, the cellular conditions and the signals that control one or other degradation pathway could be different, and are not yet known.

More than 10 years ago, it was observed that MDM2 preferentially interacts with the hypophosphorylated form of RB [29, 42]. In 2011, the team of Harbour showed that indeed, MDM2 interacts with a p38MAPK-dependent phosphorylated RB protein in Ser567 that is independent of Cdk phosphorylated RB sites [43]. p38MAPK is activated in response to different cellular stresses, such as DNA damage, osmotic shock, inflammatory response, and heat shock. After DNA damage, p38MAPK is activated in an ATM-dependent manner via Serine/threonine-protein kinase TAO involved in cell cycle regulation [44]. Under these conditions it seems that the p38α isoform is able to induce Ser567-RB phosphorylation. This phosphorylation promotes the interaction of RB with MDM2 and is involved in p53-dependent apoptosis in the Mel202 cell line. Using RNAi, it was also shown that p53 is not involved in Ser567-RB phosphorylation, or even in its degradation, but the absence of p53 under this condition inhibits cell death [43].

MDM2 is thus able to interact with the two tumor suppressor proteins; the site of interaction is different and the MDM2-RB complex is indeed able to bind p53. The formation of the triple complex RB-MDM2-p53 is stronger under DNA damage conditions, when p53 is stabilized [42]. However, RB and p53 are not able to interact directly, suggesting that MDM2 is a bridge between RB and p53 [42, 45].

### MDMX in the regulation of RB and p53

MDMX is a protein paralogous to the MDM2, they share a high similarity in their RING domains. However MDMX does not have detectable E3 ubiquitin ligase activity [46–48]. The MDMX and p53 interaction has also been very well described. The N-terminal domain of MDMX interacts with the hydrophobic pocket of the N-terminal of p53 [47]. The interaction represses the transactivity of p53; moreover, even when there is no detectable p53 ubiquitination mediated by MDMX itself, its presence stabilizes MDM2 via RING-RING interaction, and is indeed essential to promoting MDM2- mediated polyubiquitination of p53 under normal cellular conditions [49, 50]. In the same way as MDM2, under genotoxic stress conditions, MDMX switches from being a negative to a positive regulator of p53. The Ser403 near to the RING domain of MDMX is ATM-dependently phosphorylated following DNA damage; this event promotes the binding of MDMX to *p53* mRNA and acts as an RNA chaperone to properly fold the *p53* mRNA, optimizing the correct recognition for MDM2; together the two proteins enhance p53 translation ensure proper cellular response [10]. Evidence supports the idea that MDM2 and MDMX are partners and work in collaboration through the formation of a heterodimer to down-regulate p53 under normal conditions [51, 52], but also to up-regulate p53 after DNA damage [53] (Table 1).

**Figure 2:**
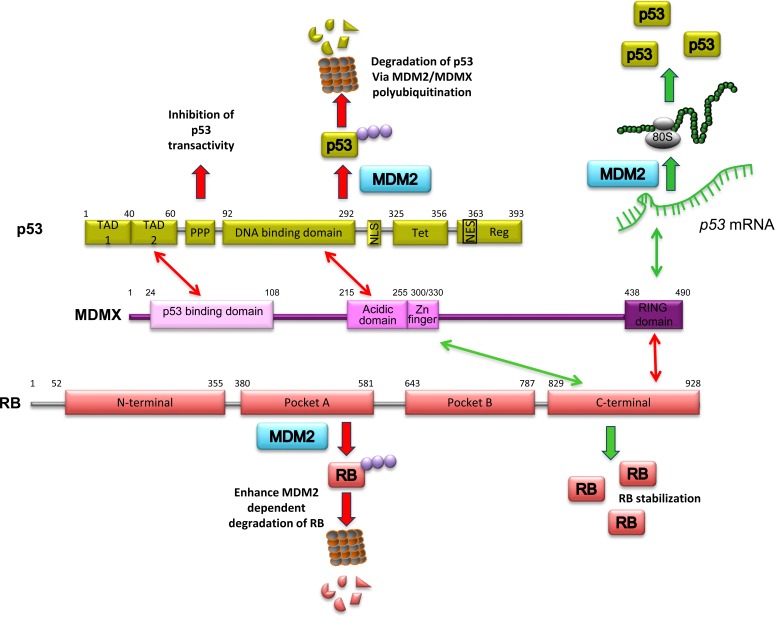
Schematic representation of MDMX, p53 and RB The different domains of each proteins are indicated, the sites of interaction between the proteins and the physiological consequences of each interaction. Note that depending on the region where RB binds to MDMX, the outcome may be MDM2-dependent RB degradation or RB stabilization.

Less is known about the MDMX and RB interplay; nonetheless, MDMX directly interacts with the RB protein. Due to the high level of identity between MDM2 and MDMX, it is not surprising that the C-terminal region in RB responsible for the recognition of MDM2 is also involved in the binding with MDMX [54]. In 2006, using the U2OS, HCT116, HEK293, and MEF cell lines, Kitagawa and colleagues, observed that the ectopic expression of MDMX inhibited RB degradation via MDM2 and stabilized RB in cells. The suggested mechanism to explain this observation is that MDMX blocks or interferes with the RB-MDM2 binding by competition with MDM2 for the C-terminal region of RB. Interestingly, a construct of MDMX that lacks the C-terminal RING domain was still able to bind RB, suggesting that the site of interaction in MDMX is apart from its C-terminal domain (Figure 2).

Using the U2OS and MEF cell lines, the direct interaction between MDMX and the C-terminal region of RB was recently corroborated. Surprisingly, in this case, the C-terminal RING domain of MDMX is involved in the binding with the C-terminal region of RB [55]. More remarkable is the fact that they observed that MDMX induces RB degradation in an MDM2-dependent manner. This effect required an MDMX-MDM2 interaction (Figure 2), whereas MDM2-mediated degradation of RB does not require the presence of MDMX [55, 56]. Accordingly, MDMX is both a negative and a positive regulator of RB, although the conditions under which each event occurs have not been described (Table 1).

### Crosstalk between RB/E2F and p53/MDM2/ MDMX pathways

Inactivation of pathways p16^Ink4a^/RB/E2F and p14^ARF^/p53/MDM2/MDMX are important mechanisms in the development of human cancer [57]. A correlation has been shown between the concomitant inactivation of both pathways and chemotherapy [58], such as in the case of some ovarian cancers [59, 60], uveal melanoma [61], malignant rhabdoid tumors [62], retinoblastoma [63], and breast cancer [58, 64].

**Figure 3:**
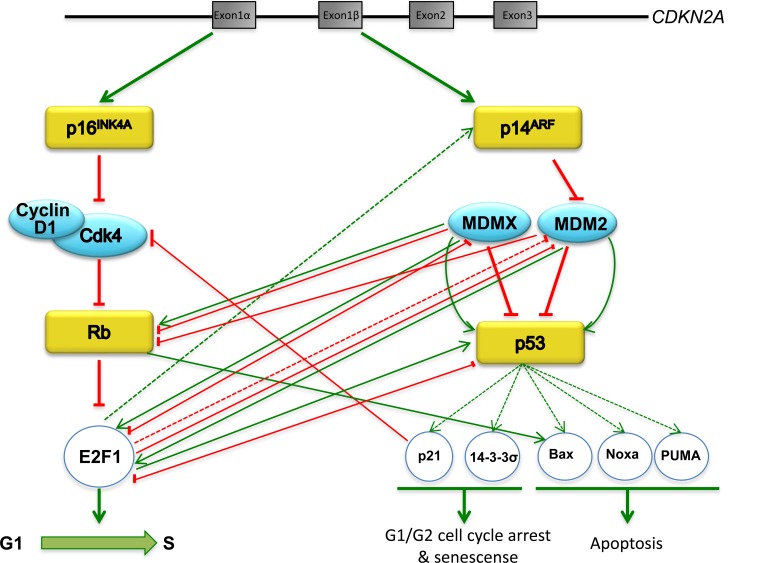
Schematic representation of the p16^Ink4a^/RB/E2F1 and p14^ARF^/p53/MDM2/MDMX pathways Tumor suppressor proteins are shown in yellow squares, proto-oncogenes are shown in blue ovals. Red lines represent negative regulation, green arrows represent positive regulation, dotted lines represent regulation at a transcriptional level.

A deficiency in the p16^Ink4a^/RB/E2F1 pathway could arise either from the loss of function of one of the two tumor suppressors RB or p16^INK4^, or from an event that allows overexpression of cyclin D1 and/or Cdk4 [65, 66] (Figure 3). Meanwhile, the p14^ARF^/p53/MDM2/MDMX pathway can be inactivated by loss of function of p53 or p14^ARF^, or by overexpression of either MDM2 or MDMX. This is exemplified in retina cancer, where 65% of tumors have an extra copy of MDMX and 10% have an extra copy of MDM2 when p53 is wild type [67–70]. The tumors with loss of RB correlate with high levels of p14^ARF^ or p53, and the explanation of this phenomenon is that in the absence of RB, E2F1 is liberated and triggers p14^ARF^ that in turn will sequester MDM2, allowing p53 to accumulate (Figure 3). However, in a scenario with overexpression of MDM2, there is no p53 accumulation, stimulating tumor growth.

Both p16^INK4^ and p14^ARF^ tumor suppressor proteins are encoded by the same locus on chromosome 9p21, through the use of two independent promoters, and encompass a unique first exon (E1α for *INK4A* and E1β for *ARF*) followed by two common exons, E2 and E3 (Figure 3). These exons are translated using different open reading frames [34, 71]. p16^INK4^ inhibits Cdk4/cyclin D1 activity and promotes RB to block cell cycle progression through the interaction with E2F1. Meanwhile, p14^ARF^ is able to bind to MDM2, preventing p53 polyubiquitination and degradation. The activation of p53 triggers p21 which causes cell cycle arrest in the G1/S phase due to suppression of Cdk/cyclin complexes. p53 could also mediate the induction of 14-3-3-σ that results in G2/M arrest [72, 73]. Through the induction of genes such as *Noxa, PUMA,* or *Bax*, p53 promotes apoptosis [1, 74]. Recently it has been shown that RB binds directly to the Bax protein at the outer membrane of the mitochondria, inducing apoptosis [14].

As mentioned above, MDM2 can also down- regulate RB, promoting cell cycle progression, or by interaction with the E2F1 transcription factor. The region of MDM2 that interacts with E2F1 is within the conserved p53-binding domain involving the amino acids 1 to 220 [75]. Given that MDM2 is an E3 ubiquitin ligase, one might expect that the principal function of this protein would be the ubiquitination of its substrates. In this particular case, MDM2 acts to prevent the ubiquitination of E2F1 by competing with the E2F1 E3 ubiquitin ligase SCF^SKP2^ [76]. In turn, E2F1 inhibits the expression of MDM2 in a p53-dependent manner [77].

In 2003, Strachan et al. described a direct interaction between E2F1 and MDMX that inhibits the function of E2F1 as a transcription factor [78]. This interaction takes place in the central portion of MDMX and near to the E2F1 DNA binding domain, between residues 117 to 241 of E2F1, with the interaction impairing the ability of E2F1 to bind DNA [78]. Thus, MDMX can affect the expression of proteins such as p53 or p14^ARF^ through the inhibition of E2F1. Moreover, an elevated expression of MDMX results in E2F1 delocalization into the cytoplasm [79]. However, it is important to note that MDMX is also able to enhance the E2F1 transactivation function through the RB degradation that results in release of E2F1 and therefore cell cycle progression [55]. Taken together, MDMX is able to enhance E2F1 function in an RB- dependent manner; but is also able to repress E2F1 activity through two different mechanisms; directly by blocking the DNA binding capacity of E2F1, and indirectly through delocalization of the transcription factor. Again, the physiological conditions that trigger one or the other are not yet known. E2F1, in turn, is able to target both MDM2 and MDMX for proteolytic degradation, in a mechanism independent of the proteasome that does not need the transcriptional activity of E2F1 [80]. Using a battery of protease inhibitors, the authors suggest that the MDMX and MDM2 degradation induced by E2F1 may be via cathepsin-like proteases, and this mechanism could also have a role in E2F1-mediated apoptosis.

Finally, we would like to discuss the direct interaction of the two transcription factors in these pathways, E2F1 and p53. In 1995, O'Connor et al. demonstrated, *in vitro* and *in vivo*, a physical interaction between the two proteins: through this interaction, p53 can suppress E2F1 transcriptional activity independently of RB [81]. Likewise, E2F1 and its partner DP1 were able to repress p53 transactivity [81, 82]. More recently, it has been shown that the cyclin A interaction domain of E2F1 is essential for the p53 binding that results in p53 stabilization and enhanced apoptosis p53-dependent under DNA damage conditions [83]. In these examples, it is clear that the same interaction can affect the function of the proteins, depending on the cellular conditions.

## CONCLUSIONS AND PERSPECTIVES

Since its discovery more than 30 years ago, the tumor suppressor protein RB has been extensively studied in a variety of cellular processes that implicate its interaction with many different partners. The best- characterized is its regulation of E2F1 and the Cdk/ cyclin complexes in cell cycle progression. However, some conflicting findings have flooded the studies of the regulation of MDM2 and MDMX on RB and other members of this pathway. Until a few years ago, MDM2 and MDMX were considered the main negative regulators of p53 [49, 84–88], but recent evidence supports the idea that they can switch to being positive regulators of p53 depending on the cellular conditions [8–11]. Taken together, these characteristics may help to explain the different and sometimes contradictory effects that have been described about the regulation of these two oncogenes on the RB pathway members. What is clear is that more knowledge is required to elucidate the mechanisms controlling the relationship of the two pathways, which may be helpful in developing programs for anticancer therapies.

## References

[1] Olivares-Illana V, Fahraeus R (2010). P53 isoforms gain functions. Oncogene.

[2] Urso L, Calabrese F, Favaretto A, Conte P, Pasello G (2016). Critical review about mdm2 in cancer: Possible role in malignant mesothelioma and implications for treatment. Crit Rev Oncol Hematol.

[3] Pasello G, Urso L, Mencoboni M, Grosso F, Ceresoli GL, Lunardi F, Vuljan SE, Bertorelle R, Sacchetto V, Ciminale V, Rea F, Favaretto A, Conte P, Calabrese F (2015). Mdm2 and hif1alpha expression levels in different histologic subtypes of malignant pleural mesothelioma: Correlation with pathological and clinical data. Oncotarget.

[4] Zhao Y, Yu H, Hu W (2014). The regulation of mdm2 oncogene and its impact on human cancers. Acta Biochim Biophys Sin (Shanghai).

[5] Zhang, Wang H (2000). Mdm2 oncogene as a novel target for human cancer therapy. Curr Pharm Des.

[6] Marine JC (2011). Mdm2 and mdmx in cancer and development. Curr Top Dev Biol.

[7] Berberich SJ (2014). Mdm2 and mdmx involvement in human cancer. Subcell Biochem.

[8] Naski N, Gajjar M, Bourougaa K, Malbert-Colas L, Fahraeus R, Candeias MM (2009). The p53 mrna-mdm2 interaction. Cell Cycle.

[9] Gajjar M, Candeias MM, Malbert-Colas L, Mazars A, Fujita J, Olivares-Illana V, Fahraeus R (2012). The p53 mrna- mdm2 interaction controls mdm2 nuclear trafficking and is required for p53 activation following DNA damage. Cancer Cell.

[10] Malbert-Colas L, Ponnuswamy A, Olivares-Illana V, Tournillon AS, Naski N, Fahraeus R (2014). Hdmx folds the nascent p53 mrna following activation by the atm kinase. Mol Cell.

[11] Candeias MM, Malbert-Colas L, Powell DJ, Daskalogianni C, Maslon MM, Naski N, Bourougaa K, Calvo F, Fahraeus R (2008). P53 mrna controls p53 activity by managing mdm2 functions. Nat Cell Biol.

[12] Weinberg RA (1995). The retinoblastoma protein and cell cycle control. Cell.

[13] Ferreira R, Naguibneva I, Pritchard LL, Ait-Si-Ali S, Harel-Bellan A (2001). The rb/chromatin connection and epigenetic control:. Opinion. Oncogene.

[14] Hilgendorf KI, Leshchiner ES, Nedelcu S, Maynard MA, Calo E, Ianari A, Walensky LD, Lees JA (2013). The retinoblastoma protein induces apoptosis directly at the mitochondria. Genes Dev.

[15] Sahi H, Savola S, Sihto H, Koljonen V, Bohling T, Knuutila S (2014). Rb1 gene in merkel cell carcinoma: Hypermethylation in all tumors and concurrent heterozygous deletions in the polyomavirus-negative subgroup. APMIS.

[16] Ohtani-Fujita N, Dryja TP, Rapaport JM, Fujita T, Matsumura S, Ozasa K, Watanabe Y, Hayashi K, Maeda K, Kinoshita S, Matsumura T, Ohnishi Y, Hotta Y (1997). Hypermethylation in the retinoblastoma gene is associated with unilateral, sporadic retinoblastoma. Cancer Genet Cytogenet.

[17] Polager S, Ginsberg D (2009). P53 and e2f: Partners in life and death. Nat Rev Cancer.

[18] Rubin SM (2013). Deciphering the retinoblastoma protein phosphorylation code. Trends Biochem Sci.

[19] Sengupta S, Henry RW (2015). Regulation of the retinoblastoma- e2f pathway by the ubiquitin-proteasome system. Biochim Biophys Acta.

[20] Wang Y, Zheng Z, Zhang J, Wang Y, Kong R, Liu J, Zhang Y, Deng H, Du X, Ke Y (2015). A novel retinoblastoma protein (rb) e3 ubiquitin ligase (nrbe3) promotes rb degradation and is transcriptionally regulated by e2f1 transcription factor. J Biol Chem.

[21] Ying H, Xiao ZX (2006). Targeting retinoblastoma protein for degradation by proteasomes. Cell Cycle.

[22] Knight JS, Sharma N, Robertson ES (2005). Epstein-barr virus latent antigen 3c can mediate the degradation of the retinoblastoma protein through an scf cellular ubiquitin ligase. Proc Natl Acad Sci U S A.

[23] Huh K, Zhou X, Hayakawa H, Cho JY, Libermann TA, Jin J, Harper JW, Munger K (2007). Human papillomavirus type 16 e7 oncoprotein associates with the cullin 2 ubiquitin ligase complex, which contributes to degradation of the retinoblastoma tumor suppressor. J Virol.

[24] Wallace M, Worrall E, Pettersson S, Hupp TR, Ball KL (2006). Dual-site regulation of mdm2 e3-ubiquitin ligase activity. Mol Cell.

[25] Picksley SM, Vojtesek B, Sparks A, Lane DP (1994). Immunochemical analysis of the interaction of p53 with mdm2;--fine mapping of the mdm2 binding site on p53 using synthetic peptides. Oncogene.

[26] Yu GW, Rudiger S, Veprintsev D, Freund S, Fernandez-Fernandez MR, Fersht AR (2006). The central region of hdm2 provides a second binding site for p53. Proc Natl Acad Sci U S A.

[27] Honda R, Yasuda H (2000). Activity of mdm2, a ubiquitin ligase, toward p53 or itself is dependent on the ring finger domain of the ligase. Oncogene.

[28] Honda R, Tanaka H, Yasuda H (1997). Oncoprotein mdm2 is a ubiquitin ligase e3 for tumor suppressor p53. FEBS Lett.

[29] Xiao ZX, Chen J, Levine AJ, Modjtahedi N, Xing J, Sellers WR, Livingston DM (1995). Interaction between the retinoblastoma protein and the oncoprotein mdm2. Nature.

[30] Sdek P, Ying H, Zheng H, Margulis A, Tang X, Tian K, Xiao ZX (2004). The central acidic domain of mdm2 is critical in inhibition of retinoblastoma-mediated suppression of e2f and cell growth. J Biol Chem.

[31] Lee C, Chang JH, Lee HS, Cho Y (2002). Structural basis for the recognition of the e2f transactivation domain by the retinoblastoma tumor suppressor. Genes Dev.

[32] Uchida C, Miwa S, Kitagawa K, Hattori T, Isobe T, Otani S, Oda T, Sugimura H, Kamijo T, Ookawa K, Yasuda H, Kitagawa M (2005). Enhanced mdm2 activity inhibits prb function via ubiquitin-dependent degradation. EMBO J.

[33] Zhang Y, Xiong Y, Yarbrough WG (1998). Arf promotes mdm2 degradation and stabilizes p53: Arf-ink4a locus deletion impairs both the rb and p53 tumor suppression pathways. Cell.

[34] Stott FJ, Bates S, James MC, McConnell BB, Starborg M, Brookes S, Palmero I, Ryan K, Hara E, Vousden KH, Peters G (1998). The alternative product from the human cdkn2a locus, p14(arf), participates in a regulatory feedback loop with p53 and mdm2. EMBO J.

[35] Sdek P, Ying H, Chang DL, Qiu W, Zheng H, Touitou R, Allday MJ, Xiao ZX (2005). Mdm2 promotes proteasome- dependent ubiquitin-independent degradation of retinoblastoma protein. Mol Cell.

[36] Amador V, Ge S, Santamaria PG, Guardavaccaro D, Pagano M (2007). Apc/c(cdc20) controls the ubiquitin- mediated degradation of p21 in prometaphase. Mol Cell.

[37] Bornstein G, Bloom J, Sitry-Shevah D, Nakayama K, Pagano M, Hershko A (2003). Role of the scfskp2 ubiquitin ligase in the degradation of p21cip1 in s phase. J Biol Chem.

[38] Jin Y, Lee H, Zeng SX, Dai MS, Lu H (2003). Mdm2 promotes p21waf1/cip1 proteasomal turnover independently of ubiquitylation. EMBO J.

[39] Kim Y, Starostina NG, Kipreos ET (2008). The crl4cdt2 ubiquitin ligase targets the degradation of p21cip1 to control replication licensing. Genes Dev.

[40] Tsvetkov P, Reuven N, Shaul Y (2010). Ubiquitin-independent p53 proteasomal degradation. Cell Death Differ.

[41] Chao CC (2015). Mechanisms of p53 degradation. Clin Chim Acta.

[42] Hsieh JK, Chan FS, O'Connor DJ, Mittnacht S, Zhong S, Lu X (1999). Rb regulates the stability and the apoptotic function of p53 via mdm2. Mol Cell.

[43] Delston RB, Matatall KA, Sun Y, Onken MD, Harbour JW (2011). P38 phosphorylates rb on ser567 by a novel, cell cycle- independent mechanism that triggers rb-hdm2 interaction and apoptosis. Oncogene.

[44] Raman M, Earnest S, Zhang K, Zhao Y, Cobb MH (2007). Tao kinases mediate activation of p38 in response to DNA damage. EMBO J.

[45] Yap DB, Hsieh JK, Chan FS, Lu X (1999). Mdm2: A bridge over the two tumour suppressors, p53 and rb. Oncogene.

[46] Stad R, Little NA, Xirodimas DP, Frenk R, van der Eb AJ, Lane DP, Saville MK, Jochemsen AG (2001). Mdmx stabilizes p53 and mdm2 via two distinct mechanisms. EMBO Rep.

[47] Shvarts A, Steegenga WT, Riteco N, van Laar T, Dekker P, Bazuine M, van Ham RC, van der Houven van Oordt W, Hateboer G, van der Eb AJ, Jochemsen AG (1996). Mdmx: A novel p53-binding protein with some functional properties of mdm2. EMBO J.

[48] Sharp DA, Kratowicz SA, Sank MJ, George DL (1999). Stabilization of the mdm2 oncoprotein by interaction with the structurally related mdmx protein. J Biol Chem.

[49] Gu J, Kawai H, Nie L, Kitao H, Wiederschain D, Jochemsen AG, Parant J, Lozano G, Yuan ZM (2002). Mutual dependence of mdm2 and mdmx in their functional inactivation of p53. J Biol Chem.

[50] Wang X, Wang J, Jiang X (2011). Mdmx protein is essential for mdm2 protein-mediated p53 polyubiquitination. J Biol Chem.

[51] Linke K, Mace PD, Smith CA, Vaux DL, Silke J, Day CL (2008). Structure of the mdm2/mdmx ring domain heterodimer reveals dimerization is required for their ubiquitylation in trans. Cell Death Differ.

[52] Tanimura S, Ohtsuka S, Mitsui K, Shirouzu K, Yoshimura A, Ohtsubo M (1999). Mdm2 interacts with mdmx through their ring finger domains. FEBS Lett.

[53] Medina-Medina I, Garcia-Beltran P, de la Mora-de la Mora I, Oria-Hernandez J, Millot G, Fahraeus R, Reyes-Vivas H, Sampedro JG, Olivares-Illana V (2016). Allosteric interactions by p53 mrna governs hdm2 e3 ubiquitin ligase specificity under different conditions. Mol Cell Biol.

[54] Uchida C, Miwa S, Isobe T, Kitagawa K, Hattori T, Oda T, Yasuda H, Kitagawa M (2006). Effects of mdmx on mdm2-mediated downregulation of prb. FEBS Lett.

[55] Zhang H, Hu L, Qiu W, Deng T, Zhang Y, Bergholz J, Xiao ZX (2015). Mdmx exerts its oncogenic activity via suppression of retinoblastoma protein. Oncogene.

[56] Hu L, Zhang H, Bergholz J, Sun S, Xiao ZX (2016). Mdm2/mdmx: Master negative regulators for p53 and rb. Mol Cell Oncol.

[57] Vogelstein B, Kinzler KW (2004). Cancer genes and the pathways they control. Nat Med.

[58] Knappskog S, Berge EO, Chrisanthar R, Geisler S, Staalesen V, Leirvaag B, Yndestad S, de Faveri E, Karlsen BO, Wedge DC, Akslen LA, Lilleng PK, Lokkevik E (2015). Concomitant inactivation of the p53- and prb- functional pathways predicts resistance to DNA damaging drugs in breast cancer in vivo. Mol Oncol.

[59] Hashiguchi Y, Tsuda H, Yamamoto K, Inoue T, Ishiko O, Ogita S (2001). Combined analysis of p53 and rb pathways in epithelial ovarian cancer. Hum Pathol.

[60] Udayakumar T, Shareef MM, Diaz DA, Ahmed MM, Pollack A (2010). The e2f1/rb and p53/mdm2 pathways in DNA repair and apoptosis: Understanding the crosstalk to develop novel strategies for prostate cancer radiotherapy. Semin Radiat Oncol.

[61] Brantley MA, Harbour JW (2000). Deregulation of the rb and p53 pathways in uveal melanoma. Am J Pathol.

[62] Venneti S, Le P, Martinez D, Eaton KW, Shyam N, Jordan-Sciutto KL, Pawel B, Biegel JA, Judkins AR (2011). P16ink4a and p14arf tumor suppressor pathways are deregulated in malignant rhabdoid tumors. J Neuropathol Exp Neurol.

[63] Ayrault O, Zindy F, Roussel MF (2007). [tp53 and rb tumor suppressor pathways collaborate in retinoblastoma genesis]. Med Sci (Paris).

[64] Knudsen ES, McClendon AK, Franco J, Ertel A, Fortina P, Witkiewicz AK (2015). Rb loss contributes to aggressive tumor phenotypes in myc-driven triple negative breast cancer. Cell Cycle.

[65] Ortega S, Malumbres M, Barbacid M (2002). Cyclin d-dependent kinases, ink4 inhibitors and cancer. Biochim Biophys Acta.

[66] Choi YJ, Anders L (2014). Signaling through cyclin d-dependent kinases. Oncogene.

[67] Guo Y, Pajovic S, Gallie BL (2008). Expression of p14arf, mdm2, and mdm4 in human retinoblastoma. Biochem Biophys Res Commun.

[68] Laurie NA, Donovan SL, Shih CS, Zhang J, Mills N, Fuller C, Teunisse A, Lam S, Ramos Y, Mohan A, Johnson D, Wilson M, Rodriguez-Galindo C (2006). Inactivation of the p53pathway in retinoblastoma. Nature.

[69] Laurie NA, Shih CS, Dyer MA (2007). Targeting mdm2 and mdmx in retinoblastoma. Curr Cancer Drug Targets.

[70] McEvoy J, Ulyanov A, Brennan R, Wu G, Pounds S, Zhang J, Dyer MA (2012). Analysis of mdm2 and mdm4 single nucleotide polymorphisms, mrna splicing and protein expression in retinoblastoma. PLoS One.

[71] Xing EP, Nie Y, Song Y, Yang GY, Cai YC, Wang LD, Yang CS (1999). Mechanisms of inactivation of p14arf, p15ink4b, and p16ink4a genes in human esophageal squamous cell carcinoma. Clin Cancer Res.

[72] Hermeking H, Lengauer C, Polyak K, He TC, Zhang L, Thiagalingam S, Kinzler KW, Vogelstein B (1997). 14-3-3 sigma is a p53-regulated inhibitor of g2/m progression. Mol Cell.

[73] Mlynarczyk C, Fahraeus R (2014). Endoplasmic reticulum stress sensitizes cells to DNA damage-induced apoptosis through p53-dependent suppression of p21(cdkn1a). Nat Commun.

[74] Chipuk JE, Kuwana T, Bouchier-Hayes L, Droin NM, Newmeyer DD, Schuler M, Green DR (2004). Direct activation of bax by p53 mediates mitochondrial membrane permeabilization and apoptosis. Science.

[75] Martin K, Trouche D, Hagemeier C, Sorensen TS, La Thangue NB, Kouzarides T (1995). Stimulation of e2f1/dp1 transcriptional activity by mdm2 oncoprotein. Nature.

[76] Zhang Z, Wang H, Li M, Rayburn ER, Agrawal S, Zhang R (2005). Stabilization of e2f1 protein by mdm2 through the e2f1 ubiquitination pathway. Oncogene.

[77] Tian X, Chen Y, Hu W, Wu M (2011). E2f1 inhibits mdm2 expression in a p53-dependent manner. Cell Signal.

[78] Strachan GD, Jordan-Sciutto KL, Rallapalli R, Tuan RS, Hall DJ (2003). The e2f-1 transcription factor is negatively regulated by its interaction with the mdmx protein. J Cell Biochem.

[79] Wunderlich M, Ghosh M, Weghorst K, Berberich SJ (2004). Mdmx represses e2f1 transactivation. Cell Cycle.

[80] Strachan GD, Rallapalli R, Pucci B, Lafond TP, Hall DJ (2001). A transcriptionally inactive e2f-1 targets the mdm family of proteins for proteolytic degradation. J Biol Chem.

[81] O'Connor DJ, Lam EW, Griffin S, Zhong S, Leighton LC, Burbidge SA, Lu X (1995). Physical and functional interactions between p53 and cell cycle co-operating transcription factors, e2f1 and dp1. EMBO J.

[82] Taura M, Suico MA, Fukuda R, Koga T, Shuto T, Sato T, Morino-Koga S, Okada S, Kai H (2011). Mef/elf4 transactivation by e2f1 is inhibited by p53. Nucleic Acids Res.

[83] Hsieh JK, Yap D, O'Connor DJ, Fogal V, Fallis L, Chan F, Zhong S, Lu X (2002). Novel function of the cyclin a binding site of e2f in regulating p53-induced apoptosis in response to DNA damage. Mol Cell Biol.

[84] Oliner JD, Kinzler KW, Meltzer PS, George DL, Vogelstein B (1992). Amplification of a gene encoding a p53-associated protein in human sarcomas. Nature.

[85] Toledo F, Krummel KA, Lee CJ, Liu CW, Rodewald LW, Tang M, Wahl GM (2006). A mouse p53 mutant lacking the proline-rich domain rescues mdm4 deficiency and provides insight into the mdm2-mdm4-p53 regulatory network. Cancer Cell.

[86] Wang SP, Wang WL, Chang YL, Wu CT, Chao YC, Kao SH, Yuan A, Lin CW, Yang SC, Chan WK, Li KC, Hong TM, Yang PC (2009). P53 controls cancer cell invasion by inducing the mdm2-mediated degradation of slug. Nat Cell Biol.

[87] Wade M, Wang YV, Wahl GM (2010). The p53 orchestra: Mdm2 and mdmx set the tone. Trends Cell Biol.

[88] Riemenschneider MJ, Buschges R, Wolter M, Reifenberger J, Bostrom J, Kraus JA, Schlegel U, Reifenberger G (1999). Amplification and overexpression of the mdm4 (mdmx) gene from 1q32 in a subset of malignant gliomas without tp53 mutation or mdm2 amplification. Cancer Res.

[89] Momand J, Zambetti GP, Olson DC, George D, Levine AJ (1992). The mdm-2 oncogene product forms a complex with the p53 protein and inhibits p53-mediated transactivation. Cell.

[90] Shimizu H, Burch LR, Smith AJ, Dornan D, Wallace M, Ball KL, Hupp TR (2002). The conformationally flexible s9-s10 linker region in the core domain of p53 contains a novel mdm2 binding site whose mutation increases ubiquitination of p53 in vivo. J Biol Chem.

[91] Finch RA, Donoviel DB, Potter D, Shi M, Fan A, Freed DD, Wang CY, Zambrowicz BP, Ramirez-Solis R, Sands AT, Zhang N (2002). Mdmx is a negative regulator of p53 activity in vivo. Cancer Res.

[92] Wei X, Wu S, Song T, Chen L, Gao M, Borcherds W, Daughdrill GW, Chen J (2016). Secondary interaction between mdmx and p53 core domain inhibits p53 DNA binding. Proc Natl Acad Sci U S A.

